# Mitochondrial function and bioenergetic trade‐offs during lactation in the house mouse (*Mus musculus*)

**DOI:** 10.1002/ece3.2817

**Published:** 2017-03-23

**Authors:** Annelise V. Mowry, Zachary S. Donoviel, Andreas N. Kavazis, Wendy R. Hood

**Affiliations:** ^1^ Department of Biological SciencesAuburn UniversityAuburnALUSA; ^2^ School of KinesiologyAuburn UniversityAuburnALUSA

**Keywords:** antioxidants, bioenergetics, house mouse, lactation, mitochondria, oxidative damage, trade‐offs

## Abstract

Energy allocation theory predicts that a lactating female should alter the energetic demands of its organ systems in a manner that maximizes nutrient allocation to reproduction while reducing nutrient use for tasks that are not vital to immediate survival. We posit that organ‐specific plasticity in the function of mitochondria plays a key role in mediating these energetic trade‐offs. The goal of this project was to evaluate mitochondrial changes that occur in response to lactation in two of the most energetically demanding organs in the body of a rodent, the liver and skeletal muscle. This work was conducted in wild‐derived house mice (*Mus musculus*) kept in seminatural enclosures that allow the mice to maintain a natural social structure and move within a home range size typical of wild mice. Tissues were collected from females at peak lactation and from age‐matched nonreproductive females. Mitochondrial respiration, oxidative damage, antioxidant, PGC‐1α, and uncoupling protein levels were compared between lactating and nonreproductive females. Our findings suggest that both liver and skeletal muscle downregulate specific antioxidant proteins during lactation. The liver, but not skeletal muscle, of lactating females displayed higher oxidative damage than nonreproductive females. The liver mass of lactating females increased, but the liver displayed no change in mitochondrial respiratory control ratio. Skeletal muscle mass and mitochondrial respiratory control ratio were not different between groups. However, the respiratory function of skeletal muscle did vary among lactating females as a function of stage of concurrent pregnancy, litter size, and mass of the mammary glands. The observed changes are predicted to increase the efficiency of skeletal muscle mitochondria, reducing the substrate demands of skeletal muscle during lactation. Differences between our results and prior studies highlight the role that an animals’ social and physical environment could play in how it adapts to the energetic demands of reproduction.

## 1 Introduction

In many species of mammals, lactation is the most energetically demanding activity that a female experiences (Speakman, 2008). To meet the demand, females are thought to partition energy resources in a manner that maximizes milk production, while reducing energy allocated to less immediate demands (Speakman, 2008). This hypothesis of energetic trade‐offs is supported by the observation that physical activity is reduced and immune responsiveness is suppressed in lactating females (Fernández‐Carmona et al., 2005; Fló, Elias, Massouh, & Roux, 1994; Medina, Smithson, & Kincade, 1993; Slonaker, 1925; Speakman, 2008; Speakman, Gidney, Bett, Mitchell, & Johnson, 2001). In addition, reduced allocation to the demands of tissue maintenance during reproduction has been purported to underlie a trade‐off between relative reproductive effort and longevity (Flatt & Heyland, 2011; Speakman & Garratt, 2014; Zera & Harshman, 2001).

While the outcomes of these energetic trade‐offs have been quantified, we know little about the cellular mechanisms that could link these processes. Several mitochondrial processes can be altered in response to a change in the metabolic demands of an organ. As mitochondria are the main source of ATP production that support the energetic demands of cells and organs, adjustments to mitochondrial efficiency determine the relative amount of substrate required to produce that ATP (Rolfe & Brown, 1997). A representative measure of the functional and coupled state of mitochondria is the respiratory control ratio (RCR) (Zhang & Hood, 2016). RCR is a valuable measure of mitochondrial respiratory ability because it represents the ability of the electron transport system (ETS) to respond to available ADP, specifically quantifying the ETS's performance (Brand & Nicholls, 2011). RCR drops with any reduction in the functional capacity of the ETS complexes (Brand & Nicholls, 2011). Cells and organs may compensate for a drop in RCR by increasing the number of mitochondria present through mitochondrial biogenesis (Pichaud, Garratt, Ballard, & Brooks, 2013). In contrast, the P/O ratio targets the coupling efficiency of the mitochondria and substrate oxidation (Salin, Auer, Rey, Selman, & Metcalfe, 2015; Zhang & Hood, 2016). Change in P/O reflects changes in the proton motive force across the inner mitochondrial membrane due to a change in the activity of uncoupling proteins, adenine nucleotide transporters, and the fatty acid content of the lipid bilayer. In addition, P/O is altered by the substrate being used to support ATP production. A change in substrate oxidation in one organ may alter the availability of substrate for another organ, supporting trade‐offs in metabolic function (Salin et al., 2015).

Organ‐specific changes or adjustments in mitochondrial function can influence the production of reactive oxygen species (ROS) (Jastroch, Divakaruni, Mookerjee, Treberg, & Brand, 2010; Ricquier & Bouillaud, 2000). ROS are created, in part, when electrons react with molecular oxygen to form a superoxide. ROS production can occur at 11 different points within the ETS, but most is generated by complex I and III (Brand, 2000, 2016; Brand & Nicholls, 2011; Brand et al., 2004; Murphy, 2009; Ricquier & Bouillaud, 2000; Speakman et al., 2004). In general, the slower the electrons move through the ETS, the greater the chance they will form ROS (Brand, 2000; Jastroch et al., 2010; Murphy, 2009; Ricquier & Bouillaud, 2000). To neutralize ROS and prevent damage, mitochondria and cells can produce antioxidants and upregulate the expression of uncoupling proteins (UCPs). Production of antioxidants is thought to be energetically expensive, as is protein synthesis related to mitochondrial biogenesis (Lane & Martin, 2010). Organisms may reduce ROS production by increasing the expression of UCPs (Brand, 2000). UCPs appear to provide a relatively inexpensive way to regulate energy production and maintain control over the inner mitochondrial membrane and reactive oxygen species (ROS) production (Brand, 2000). The expression of UCPs increases proton leak across the inner mitochondrial membrane, uncoupling proton movement and ATP synthesis. Protein synthesis makes up almost 75% of a cell's energy expenditure (Lane & Martin, 2010). Each mitochondrion requires multiple expression of almost 600 genes (Mootha et al., 2003), and the half‐life for a population of mitochondria is 2 days to 2 weeks (Gottlieb & Stotland, 2015; Lipsky & Pedersen, 1981; Menzies & Gold, 1971). Thus, organ‐specific changes to mitochondrial biogenesis and antioxidant production is an energetically expensive endeavor.

Numerous physiological changes occur throughout the body to support the initial demands of pregnancy and, later, the demands of lactation (Akers, 2002). Apart from the obvious changes to the uterus and mammary glands, other energetically demanding organs also display considerable plasticity during a reproductive event. The liver and skeletal muscles are the most demanding organs within in the body, contributing to about 50% of the body's basal metabolic rate in a nonreproductive individual (Rolfe & Brown, 1997). Both of these organs are known to display bioenergetic changes during reproduction. For example, Pichaud, Garratt, et al., (2013) showed that the liver of wild‐derived house mice housed in standard laboratory boxes display increased mitochondrial density, antioxidant capacity, and oxidative damage, but no change in RCR. An increase in liver size, mitochondrial density, and antioxidant capacity undoubtedly play a critical role in the synthesis of fatty acids used by the mammary gland as substrate for the production of triacylglycerols (Vernon, 1989), which are abundant in mouse milk (Knight, Maltz, & Docherty, 1986). In contrast, mitochondria of skeletal muscle collected from laboratory mice housed under similar conditions downregulated the expression of mRNA for proteins involved in glycolysis, the citric acid cycle, and lipid metabolism. In addition, uncoupling protein 3 was downregulated. Collectively, these would result in reduced ATP production and reduced amino acid and lipid use by skeletal muscle (Xiao, Grove, Grayson, & Smith, 2004; Xiao, Grove, & Smith, 2004). Together, these studies on the bioenergetic function of the liver and muscle provide evidence that different organs have different responses to reproduction. Moreover, the bioenergetic demands and responses of organs are plastic, and they may vary between species and strains and with the relative activity and environment of the animal. Indeed, measurements of oxidative damage within the liver of reproductive birds and mammals show an upregulation in some species and a downregulation in others (Blount, Vitikainen, Stott, & Cant, 2016; Speakman & Garratt, 2014). With this investigation, we simultaneously measure mitochondrial function in skeletal muscle and liver of wild‐derived house mice living in seminatural enclosures to assess the metabolic adjustments to lactation that females are likely to make under natural conditions.

Because the liver and the skeletal muscle have the greatest potential to compete for the demands of the mammary gland, we posit that how the liver and skeletal muscle mitochondria respond to reproduction will play a formative role in determining how much energy can be allocated to a reproductive event. The first step in testing this hypothesis is to evaluate mitochondrial processes in the liver and skeletal muscle of lactating and nonreproductive mice to determine how mitochondrial function varies between organs within the same individual. The mass of the liver increases during reproduction, but mouse activity levels drop and skeletal muscle mass remains unchanged (Speakman, 2008; Williamson, 1980). We predict that lactating females will upregulate the respiratory function of the liver while downregulating the respiratory capacity of skeletal muscle. These changes could include an increase (liver) or decrease (skeletal muscle) in organ size, mitochondrial density, and mitochondrial respiratory capacity (RCR and P/O). Both organs are predicted to adopt measures that will reduce oxidative damage, reducing the demand for lipid, protein, and DNA repair. To reduce oxidative damage, antioxidant levels and the expression of RNA coding for proteins that support mitochondrial uncoupling are predicted to increase. A decrease in the demand for substrate for respiration by skeletal muscle and reduced oxidative damage in both the liver and skeletal muscle should spare substrate for the high demands of mammary function. In addition, mitochondrial function in the liver and skeletal muscle are expected to vary with measures of relative allocation to reproduction such as mammary mass, number of offspring, and stage of concurrent pregnancy. These relationships will also be evaluated.

## 2 Materials and Methods

### 2.1 Study animals

We evaluated mitochondrial function, oxidative damage, and antioxidant levels in house mice, *Mus musculus*. The mice used in this study are descended from individuals that were obtained from Dr. Wayne Potts at the University of Utah. These outbred lines of house mice were originally collected in Gainesville, FL, USA. Animals included in this study were 14 generations removed from the wild and were in enclosures designed to mimic the natural environment of wild mice. At generation 11, the mice had an average relatedness coefficient of .0839, representing a 12.8% reduction in initial heterozygosity (Gaukler et al., 2016). During generations 12–14, mice were maintained in the seminatural populations in Auburn. New generations were founded with individuals from different enclosures, but parentage of all animals included in this study was not available to recalculate heterozygosity.

### 2.2 Experimental design

In the wild, mice live in social groups, referred to as demes, which are typically comprised of seven to twelve adults. Each adult maintains a home range size of approximately a few meters (Klein, 1975). For this study, mice were maintained in enclosures designed to simulate these natural conditions. Four, 5‐m^2^ enclosures were divided between two secure buildings, each with a roof and hardware‐cloth windows to excluded predators while exposing the mice to ambient temperatures, noise, and seasonal weather conditions. All enclosures were within 6 m of each other and thus experienced similar ambient conditions, although small differences in microclimate were possible. All animals were offered ad libitum access to water and a rodent chow diet (Teklad Global Diet 2019). All adult mice were pit‐tagged (model number HPT12, Biomark, Inc., Boise, ID, USA) and ear punched for identification purposes.

At sexual maturity (~2 months of age), each reproductive population was created with three male and five female mice. Within each population's enclosure, mice were allowed to maintain a natural social structure and breed at a natural pace. Mice were offered eight terracotta pots (four in tall, four in diameter) with an opening added to the side and saucers on top for lids for nesting. The small size of these pots limited communal nesting and suckling that could alter the energetic output of reproduction. Litters and putative mothers were carefully monitored by daily census. Pups were removed at peak lactation (~day 14) to maintain population densities and encourage rapid reproductive turnover. In mice and many other species, primiparous mothers often allocate less to reproduction than experienced females (Fuchs, 1982). Thus to limit data to females expected to maximize their allocation to reproduction, we allowed all reproductive females to breed and produce three to five successful litters before organs were collected. Litters were deemed successful when at least two pups survived to peak lactation (day 14). Five females did not have three successful litters and thus were not included in the final sample of lactating animals (number included = 10). Females and their litter were sacrificed fourteen days postpartum. All nonreproductive females (*n* = 7) were maintained in a single enclosure. Nonreproductive controls were sacrificed in an age‐matched manner to control for effects associated with age.

### 2.3 Tissue collection

Females were killed using isoflurane vapors, weighed, and decapitated. Immediately upon decapitation, the liver and hindlimb skeletal muscles were consistently extracted and weighed. A sample of the liver (approximately 0.5 g) and skeletal muscle (hindlimb muscles, approximately 0.3 g) was used for mitochondrial isolation, as described below. A second sample of liver (approximately 0.75 g) and skeletal muscle (hindlimb, approximately 0.3 g) was collected and flash‐frozen in liquid nitrogen and stored at −80°C for subsequent analysis. Mice often mate and become pregnant while lactating. Thus, at dissection, the stage of pregnancy of each female was classified as early, mid‐ or late pregnancy based on fetal size. Early pregnancy was indicated with the presence of underdeveloped embryos that were just a few millimeters long, mid‐pregnancy when fetuses were approximately 5 mm long with obvious limbs and underdeveloped facial features, and late pregnancy when fetuses displayed fully developed limbs and well‐developed face. The left mammary gland was also extracted and weighed as an indication of relative allocation to milk production.

### 2.4 Mitochondria isolation

Mitochondrial isolation was performed using differential centrifugation by utilizing previously published techniques (Makinen & Lee, 1968; Sewell, Wostmann, Gairola, & Aleem, 1975). Briefly, livers were weighed and put into 10 volumes of a solution made up of 250 mmol/L sucrose, 5 mmol/L HEPES, and 1 mmol/L EGTA and minced with scissors. This minced tissue was further homogenized with a Potter‐Elvhjem PTFE pestle and glass tube. The resulting homogenate was centrifuged for 10 min at 500 *g* at 4°C, pelleting the cellular debris. The supernatant was then decanted through cheesecloth and then centrifuged for 10 min at 3,500 *g* at 4°C, pelleting the mitochondrial fraction. The supernatant was removed, and the pellet resuspended in the sucrose solution. This solution was centrifuged for 10 min at 3,500 *g* at 4°C, the supernatant discarded, and the final mitochondrial pellet suspended in 250 μl of a solution made up of 220 mmol/L mannitol, 70 mmol/L sucrose, 10 mmol/L Tris+HCl, and 1 mmol/L EGTA, at a pH of 7.4.

**Figure 1:**
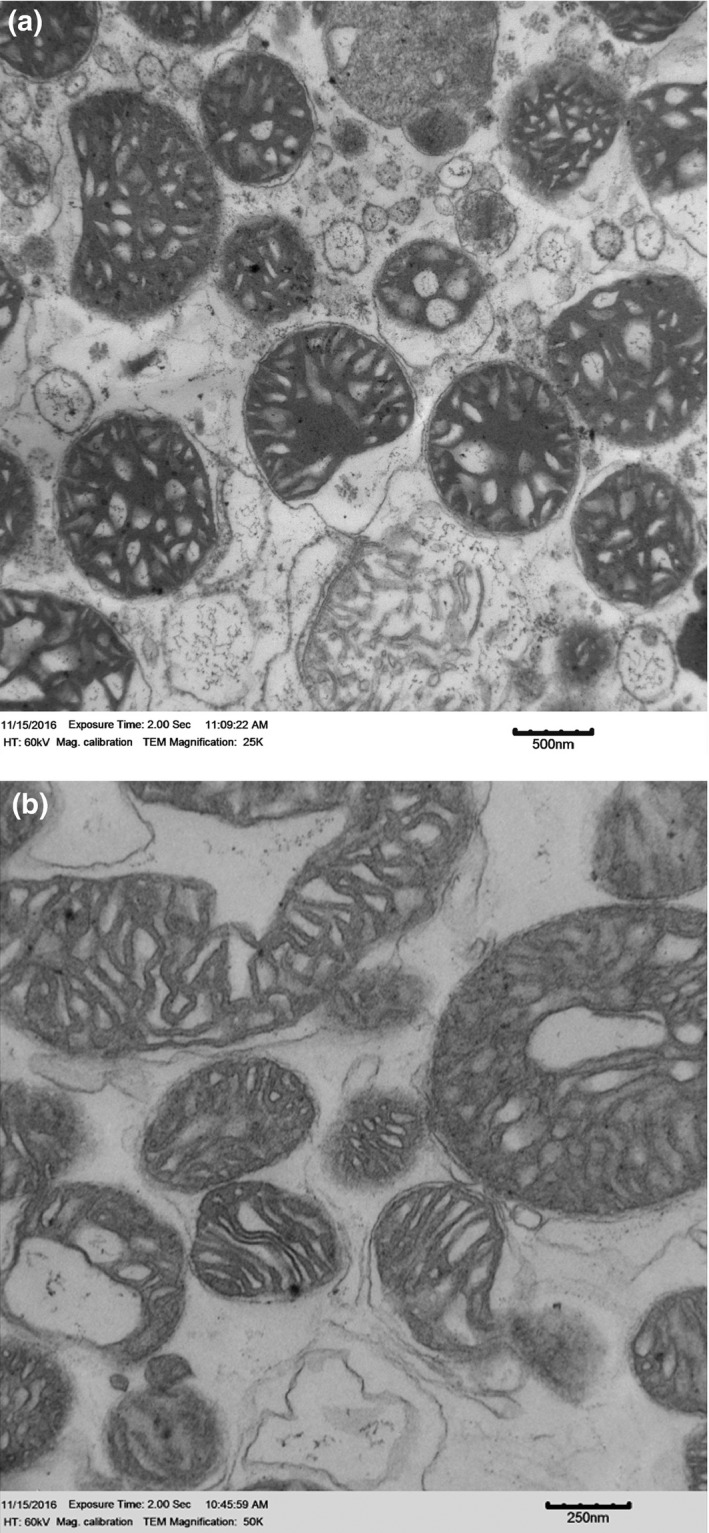
Transmission electron micrograph of isolated mitochondrial in liver (25K magnifications) (a) and skeletal muscle (b)

Excised skeletal muscles were trimmed off fat and connective tissue, weighed, and put in 10 volumes of BSA solution (100 mmol/L KCl, 40 mmol/L Tris‐HCl, 10 mmol/L Tris‐base, 1 mmol/L MgSO4, 0.1 mmol/L EDTA, 0.2 mmol/L ATP, and 2% (wt/vol) free fatty acid bovine serum albumin, pH 7.40). Muscles were minced with scissors and then homogenized for 5 s with a polytron. Trypsin (5 mg/g of wet muscle) was added and mixed continually for 7 min to digest the minced muscle. This reaction was terminated by the addition of another 10 volumes of BSA solution. The homogenate was centrifuged at 500 *g* for 10 min at 4°C pelleting down cellular debris. The supernatant was decanted through cheesecloth and centrifuged at 3,500 *g* for 10 min to pellet the mitochondrial fraction. The supernatant was discarded and the remaining mitochondrial pellet resuspended in BSA solution. This was then centrifuged at 3,500 *g* for 10 min. The supernatant was discarded and the pellet was resuspended in 10 volumes of a no‐BSA solution (similar to BSA solution, but without BSA). This resuspended pellet was centrifuged at 3,500 *g* for 10 min, and the final mitochondrial pellet suspended in 250 μl of a solution made up of 220 mmol/L mannitol, 70 mmol/L sucrose, 10 mmol/L Tris+HCl, and 1 mmol/L EGTA, at a pH of 7.4. The quality of our mitochondria isolation was confirmed with transmission electron microscopy, which showed minimal evidence of mitochondrial damage for liver or skeletal muscle (Figure 1).

### 2.5 Mitochondria respiration measurements

Liver and skeletal muscle mitochondrial respiration were measured polarographically (Hansatech Instruments, Norfolk, UK) using a technique described by Messer, Jackman, and Willis (2004). Briefly, 20 μl of isolated mitochondria were placed in a total of 1 ml of respiration buffer adjusted from the solution described by Wanders, Groen, Van Roermund, and Tager (1984) (100 mmol/L KCL, 50 mmol/L MOPS, 10 mmol/L KH_2_PO_4_, 20 mmol/L glucose, 10 mmol/L MgCl2, 1 mmol/L EGTA, and 0.2% fatty acid‐free BSA; pH = 7.0) in the respiratory chamber and spun constantly at 37°C; 2 mmol/L pyruvate and 2 mmol/L malate were used as substrates. The maximal respiration (state 3), defined as the rate of respiration in the presence of ADP, was initiated by adding 0.25 mmol/L ADP to the respiration chamber containing mitochondria and respiratory substrates. State 4 respiration was recorded following the phosphorylation of ADP. State 3 and state 4 respiration rates were normalized to mitochondrial protein concentration and expressed as O_2_/mg mitochondrial protein/min. The respiratory control ratio (RCR) was calculated by dividing state 3 by state 4 respiration and the P/O (i.e., ADP phosphorylated per oxygen atom consumed) was determined (Estabrook, 1967).

### 2.6 RNA isolation, reverse transcription, and RT‐PCR

Whole liver and skeletal muscle tissue were homogenized with a polytron homogenizer in Ribozol. The sample was vortexed and centrifuged at 13,000 *g* for 10 min at 4°C. The supernatant was transferred to a new tube and mixed with chloroform before being centrifuged at 13,000 *g* for 15 min at 4°C. The aqueous phase was transferred to a new tube, and RNA was precipitated with isopropanol and pelleted out using centrifugation and then washed twice with two volumes of 75% ethanol. The pellet was resuspended in RNase‐free water. The concentration was measured using NanoDrop Lite Spectrophotometer (Thermo Fisher Scientific, Waltham, MA, USA). Total RNA was stored at −80°C.

cDNA was synthesized with 1 μg RNA, 4 μl qScript, and DEPC water. The mixture was incubated at 25°C for 5 min, 42°C for 30 min, 85°C for 5 min, and then held at 4°C. The cDNA was diluted to a final concentration of 5 ng/μl. Quantitative real‐time PCR was performed by SYBR green chemistry (Quanta BioSciences, Gaithersburg, MD, USA) using CFX Connect™ Real‐Time PCR Detection System (Bio‐Rad Laboratories, Hercules, CA, USA). Primers and probes for uncoupling protein 2 (UCP2; forward primer 5′‐CCTCCCCTGTTGATGTGGTC‐3′ and reverse primer 5′‐GAGCATGGTAAGGGCACAGT‐3′), peroxisome proliferator‐activated receptor gamma coactivator 1‐alpha (PGC‐1α; forward primer 5′‐TCGCAGAAGCAGTGTTCCAT‐3′ and reverse primer 5′‐CCATGGTCGTATCAGAGGCC‐3′), and the reference gene beta‐actin (β‐actin; forward primer 5′‐GTGGATCAGCAAGCAGGAGT‐3′ and reverse primer 5′‐ACGCAGCTCAGTAACAGTCC‐3′) were obtained from Integrated DNA Technologies, Inc. Each 20 μl PCR reaction was performed in duplicate with a 25.0 ng cDNA template. Relative quantification of gene expression was performed using the 2∆∆CT method whereby ∆CT [i.e., CT(reference gene) − CT(gene of interest)].

### 2.7 Protein electrophoresis

Protein concentration in isolated mitochondria was measured following the technique of Bradford (Bradford, 1976a,b). Proteins (20 μg) were separated in 0.1% sodium dodecyl sulfate polyacrylamide gels by polyacrylamide gel electrophoresis. Proteins were then transferred to PVDF membranes. Nonspecific proteins were blocked using a buffer of phosphate‐buffered saline (PBS) with 0.05% Tween and 5% nonfat milk. Membranes were incubated with primary antibodies using 1:1,000 dilution (copper–zinc superoxide dismutase (CuZnSOD), manganese superoxide dismutase (MnSOD), glutathione peroxidase 1 (Gpx‐1), and catalase (GeneTex, Irvine, CA, USA)) and 4‐hydroxynonenal (4‐HNE) adducts (Abcam, Cambridge, MA, USA) for 1 hr at room temperature. Membranes were then washed thoroughly with PBS containing 0.05% Tween and incubated again with secondary antibodies using 1:2,000 dilution (GeneTex, Irvine, CA, USA) for 1 hr at room temperature and then washed again. A chemiluminescent system was used to visualize the marked proteins (GE Healthcare Life Sciences, Pittsburgh, PA, USA). Luminescent membrane images were taken and visualized with the ChemiDocIt^2^ Imaging System (UVP, LLC, Upland, CA, USA). Ponceau staining standardized sample loading. Gpx‐1 and CuZnSOD data for liver and muscle, respectively, are not presented due to technical errors and insufficient tissue to repeat analyses.

### 2.8 Statistical analysis

All statistical analyses were performed using SAS 9.4 (SAS Institute, Cary, NC, USA). Comparisons between lactating and nonreproductive groups were completed with a mixed model that included enclosure as a random effect. For lactating females, regression was used to evaluate the relationship between the mass of a mammary gland, pregnancy stage, and size of the final litter suckled and the morphological, bioenergetic, and gene expression variables included in this study. Due to limited sample size within group, we did not control for enclosure in the regression analyses. Statistical significance was established at *p* < .05. Standard errors associated with all means are given.

## 3 Results

### 3.1 Organ mass

Lactating females included in this study successfully weaned 5–13 offspring throughout their reproductive lives. The average body mass was significantly different between lactating (30.4 ± 1.4 g) and nonreproductive females (19.0 ± 1.7 g; *F*_1,14_ = 27.4, *p* < .01); Organ masses were compared between groups. Lactating females had significantly heavier livers (2.25 ± 0.30 g) than nonreproductive females (0.95 ± 0.48 g; *F*_1,14_ = 5.32, *p* = .04). This change in liver mass tracked change in body mass, as liver mass as a percent of body mass did not differ between groups (lactating = 7.41% ± 0.88%, nonreproduction = 4.95% ± 1.46%; *F*_1,14_ = 2.08, *p* = .17). In contrast, there was no difference in hindlimb muscle mass of lactating females (0.66 ± 0.05 g) and nonreproductive controls (0.66 ± 0.04 g; *F*_1,14_ = 0.03, *p* = .87). Muscle mass did not change with body mass during reproduction, as is evident by a significant decrease in hindlimb muscle mass relative to body mass (lactating = 2.19% ± 0.18%, nonreproduction = 3.53% ± 0.23%, *F*_1,14_ = 21.2, *p* < .01). The mass of the left mammary gland ranged from 0.19 to 0.81 g and the pregnancy stage of lactating females ranged from early to late, with one female displaying no signs of current pregnancy. In lactating females, the mass of the liver increased as the mass of the mammary gland increased (*F*_1,7_ = 37.1, *p* < .01, *R* ^2^ = .84).

### 3.2 Mitochondrial oxidative phosphorylation

**Figure 2:**
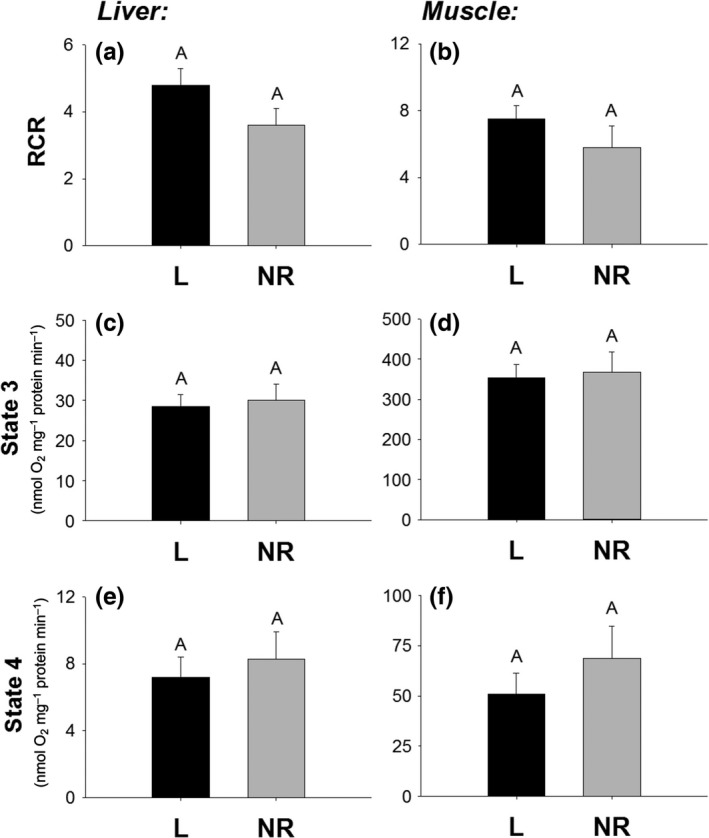
Liver RCR (a), skeletal muscle RCR (b), liver state 3 respiration (c), skeletal muscle state 3 respiration (d), liver state 4 respiration (e), and skeletal muscle state 4 respiration (f) of isolated mitochondria of lactating (L) and nonreproductive (NR) female mice. Bar graphs show means and standard error bars. Letters above bars indicate results of *t*‐test with significant differences represented by different letters. Significance established at *p* < .05

RCR was calculated from isolated mitochondria in the liver and skeletal muscle as a general measure of mitochondrial efficiency at peak lactation. RCR of liver mitochondria did not differ between lactating and nonreproductive mice (*F*_1,12_ = 2.32, *p* = .15; Figure 2a), nor did state 3 (*F*_1,13_ = 0.10, *p* = .76; Figure 2c) or state 4 respiration (*F*_1,13_ = 0.26, *p* = .62; Figure 2e). The RCR of skeletal muscle was also similar between lactating and nonreproductive mice (*F*_1,12_ = 1.35, *p* = .27; Figure 2b), as were state 3 (*F*_1,12_ = 0.06, *p* = .82; Figure 2d) and state 4 respiration (*F*_1,12_ = 0.87, *p* = .37; Figure 2f). The P/O ratio of the mitochondria did not differ between groups for the liver (lactating = 2.23 ± 0.11, nonreproductive = 2.25 ± 0.17, *F*_1,12_ = 0.01, *p* = .93,) or muscle (lactating = 2.58 ± 0.13, nonreproductive = 2.49 ± 0.21, *F*_1,13_ = 0.16, *p* = .70).

**Figure 3:**
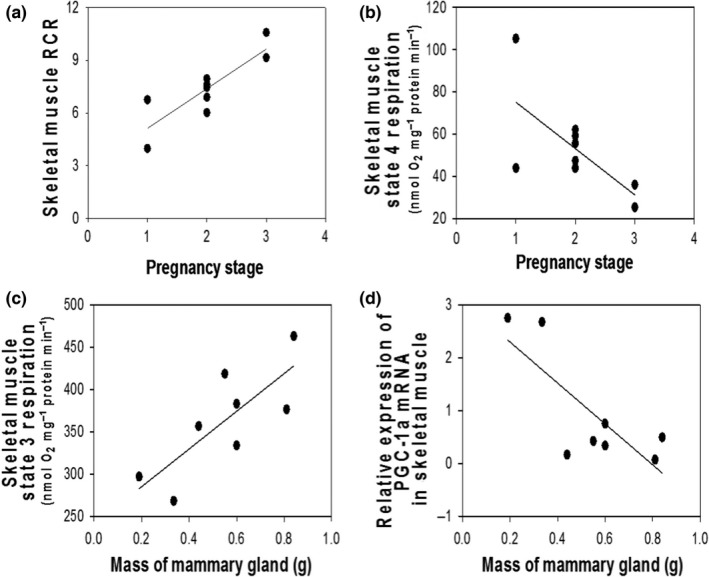
Relationships of skeletal muscle variables to pregnancy stage (a and b) or mass of the mammary gland (c and d) in lactating mice. Pregnancy stages are described in the methods section

In lactating females, the respiratory function of the mitochondria in muscle changed with both stage of pregnancy with concurrent lactation and the size of the litter that females were suckling. Muscle RCR increased, while state 4 respiration decreased as fetuses reached a later stage of development (RCR: *F*_1,7_ = 18.8, *p* < .01, *R* ^2^ = .73; Figure 3a; state 4: *F*_1,7_ = 6.21, *p* = .04, *R* ^2^ = .47; Figure 3b). State 3 respiration of the mitochondrial in the muscle increased as the mass of the mammary gland increased (*F*_1,6_ = 9.58, *p* = .02, *R* ^2^ = .61; Figure 3c) in lactating females.

### 3.3 Mitochondrial biogenesis

Peroxisome proliferator‐activated receptor gamma coactivator 1‐alpha (PGC‐1α) regulates several genes involved in energy metabolism and functions as an important regulator of mitochondrial biogenesis. To measure how the energetic demand of reproduction altered the expression of PGC‐1α, mRNA was measured. Liver and skeletal muscle displayed no difference in the relative expression of mRNA coding for PGC‐1α (Liver: lactating = 2.25 ± 0.53 and nonreproductive = 1.00 ± 0.85**,** *F*_1,12_ = 1.55, *p* = .24; muscle: lactating = 0.99 ± 0.62 and nonreproductive = 1.00 ± 1.02**,** *F*_1,12_ = 0.00, *p* = .99). But, PGC‐1α mRNA expression in skeletal muscle declined as mammary mass increased in lactating females (*F*_1,6_ = 9.28, *p* = .02, *R* ^2^ = .61; Figure 3d).

### 3.4 Antioxidants and oxidative damage

To evaluate how peak lactation affects the redox state of the tissues of interest, antioxidant levels and oxidative damage were assessed, including the antioxidants catalase, copper–zinc superoxide dismutase (CuZnSOD, i.e., SOD1), manganese superoxide dismutase (MnSOD, i.e., SOD2), and glutathione peroxidase‐1 (Gpx‐1). As a measure of oxidative damage, 4‐hydroxynonenal (4‐HNE) adducts, a product of lipid peroxidation, was quantified.

**Figure 4:**
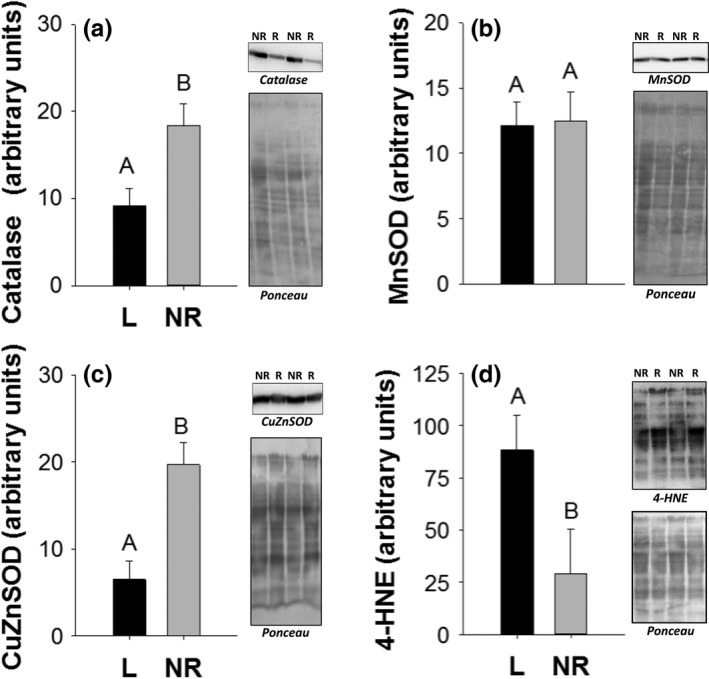
Liver catalase (a), manganese superoxide dismutase (b), copper–zinc superoxide dismutase (c), and 4‐hydroxynonenal (d) levels in arbitrary units from isolated mitochondria of lactating (L) and nonreproductive (NR) female mice. Bar graphs show means and standard error bars. Letters above bars indicate results of *t*‐test with significant differences represented by different letters. Significance established at *p* < .05. Representative Western blots are shown to the right of graphs

In the liver of lactating mice, isolated mitochondria had significantly less catalase (*F*_1,14_ = 8.67, *p* < .01; Figure 4a) and CuZnSOD (*F*_1,13_ = 15.7, *p* < .01; Figure 4c), but MnSOD levels did not differ between groups (*F*_1,12_ = 0.10, *p* = .75; Figure 4b). Levels of 4‐HNE adducts were higher in the liver mitochondria of lactating versus nonreproductive individuals (*F*_1,12_ = 4.39, *p* = .05; Figure 4d).

**Figure 5:**
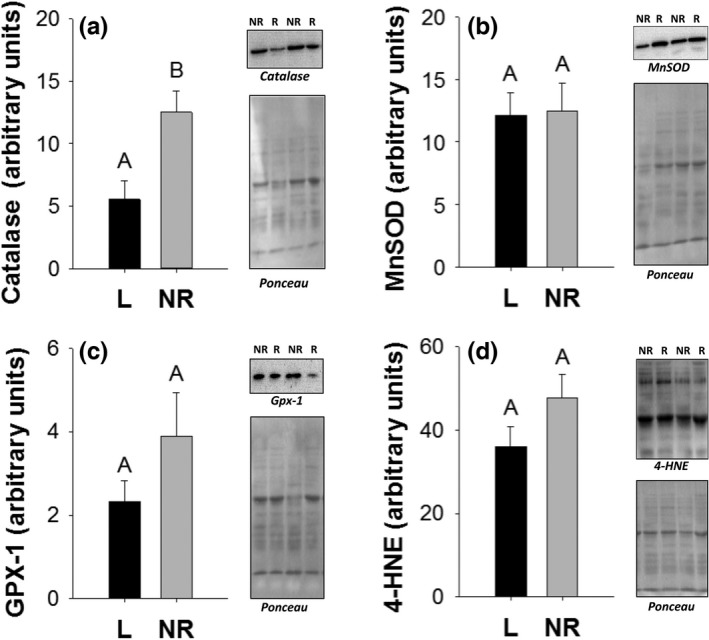
Skeletal muscle catalase (a), manganese superoxide dismutase (b), glutathione peroxidase 1 (c), and 4‐hydroxynonenal (d) levels in arbitrary units from isolated mitochondria of lactating (L) and nonreproductive (NR) female mice. Bar graphs show means and standard error bars. Letters above bars indicate results of *t*‐test with significant differences represented by different letters. Significance established at *p* < .05. Representative Western blots are shown to the right of graphs

**Figure 6:**
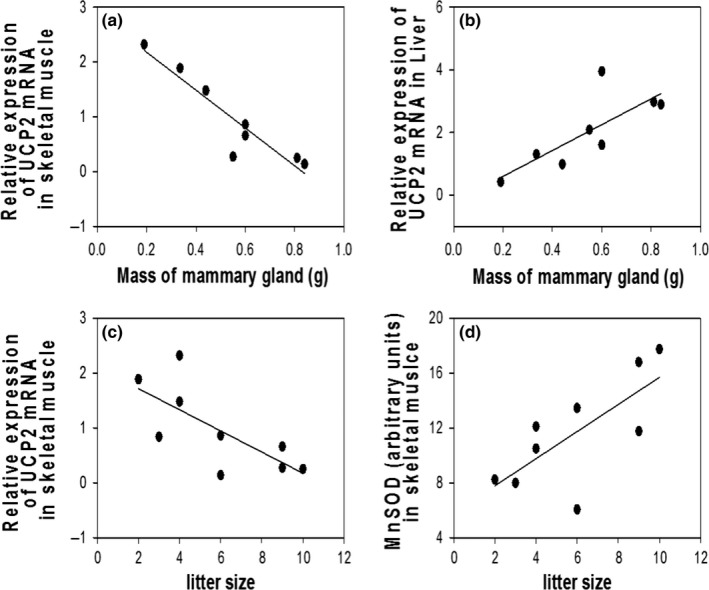
Relationships of liver (b) and skeletal muscle (a, c, d) variables to the mass of the mammary gland or litter size in lactating mice

Isolated skeletal muscle mitochondria had significantly lower catalase levels in lactating versus nonreproductive mice (*F*_1,12_ = 9.29, *p* = .01; Figure 5a). Isolated muscle mitochondria of lactating and nonreproductive mice had similar levels of MnSOD (*F*_1,13_ = 0.01, *p* = .91; Figure 5b) and Gpx‐1 (*F*_1,14_ = 1.52, *p* = .24; Figure 5c). Skeletal muscle mitochondria displayed no difference in levels of 4‐HNE adducts, between groups (*F*_1,14_ = 2.50, *p* = .14; Figure 5d). However, skeletal muscle MnSOD increased as litter size increased in lactating females (*F*_1,7_ = 7.70, *p* = .03, *R* ^2^ = .52; Figure 6d), but no other significant relationships were found between oxidative damage or antioxidants and pregnancy stage, mammary mass, or litter size.

### 3.5 Uncoupling proteins

The efficiency of energy production through oxidative phosphorylation is affected by several regulated variables, including the uncoupling of the ETS. To evaluate how the physiological demand of reproduction alters the degree of uncoupling, mRNA expression of uncoupling protein 2 (UCP2) was evaluated. There was no difference in UCP2 mRNA levels for lactating and nonreproductive mice in the liver (lactating = 2.25 ± 0.53, nonreproductive = 1.00 ± 0.85, *F*_1,12_ = 1.55, *p* = .24) or skeletal muscle (lactating = 0.96 ± 0.36, nonreproductive: 1.00 ± 0.56, *F*_1,13_ = 0.00, *p* = .96). In contrast, UCP2 mRNA in the liver increased as mammary mass increased (*F*_1,7_ = 7.70, *p* = .03, *R* ^2^ = .60; Figure 6b) but decreased in the muscle as mammary mass increased (*F*_1,7_ = 7.70, *p* = .03, *R* ^2^ = .87; Figure 6a) and litter size increased (*F*_1,7_ = 7.70, *p* = .03, *R* ^2^ = .52; Figure 6c).

## 4 Discussion

While studies evaluating the costs of reproduction clearly show that the production of offspring is associated with a modest to dramatic change in the relative energy requirements of females (Gittleman & Thompson, 1988; Oftedal, 1984; Speakman, 2008), we know relatively little about how the bioenergetic functions of organs change in response to this high demand. Our findings suggest that the liver and the skeletal muscle display similarities and differences in how they respond to the demands of lactation.

The mass of the liver for lactating females was more than double that of nonreproductive females. This change largely tracked body mass, as the body mass of lactating females was about 50% greater than the body mass of nonreproductive females, and liver mass as a percent of body mass did not differ between groups. Not surprisingly, the mass of the liver was positively correlated with the mass of the mammary gland, as a major role of the liver during lactation is to support the production of lipids that are used by the mammary gland to build triacylglycerides (Akers, 2002). RCR, state 3 and 4 respiration, P/O, and relative expression of PGC‐1α mRNA, which codes for a protein that is pivotal to the upregulation of mitochondrial biogenesis, were not different between liver of lactating and nonreproductive females. These findings suggest that the liver can support much of the change in substrate demand associated with lactation by increasing its relative size. Unlike the liver, the hindlimb musculature displayed no change in size during lactation, but there was also no change in the energetic capacity of the skeletal muscle based on RCR, state 3 and 4 respiration, P/O, and relative expression of PGC‐1α mRNA. Despite this lack of variation, regression analysis revealed that the respiratory function of mitochondria in the skeletal muscle fluctuates with the relative demand placed on lactating females.

House mice go into estrus after parturition and typically mate the night after birth when a male is present. Implantation of fertilized eggs may be immediate or delayed. The length of the delay is positively correlated with the size of the litter being suckled (Brambell, 2016; Oswald & McClure, 1987). As all females in this study were culled at day 14 of lactation, variation in relative stage of current pregnancy would have been associated with variation in relative length of a delay in implantation. The positive correlation between stage of fetal development and muscle RCR in lactating females could either be associated with a response to the compounding effect of pregnancy and lactation or it could reflect a relationship between metabolic capacity and the length of the delay, where females with the highest metabolic capacity display the shortest delay. It is interesting that this difference was apparent in the muscle, but not the liver. The relative increase in muscle RCR was driven by a decline in state 4 respiration. Efficient mitochondria idle at low state 4 respiration, consuming little O_2_ to maintain the proton motive force across the inner mitochondria membrane, while still having the ability to achieve a high maximal substrate oxidation and ATP turnover, or state 3, when necessary (Brand, 2000; Brand & Nicholls, 2011; Lambert & Brand, 2004; Ricquier & Bouillaud, 2000; Speakman et al., 2004). As lactating females experience a reduction in activity (Slonaker, 1925), their muscle mitochondria likely spend more time functioning at a level that nears state 4 respiration than nonreproductive mice. Thus, increased RCR and reduced state 4 respiration would reduce the relative damage of the muscle, sparing nutrients for other organs. This process is complicated by the fact that less proton leak increases the proton motive force, resulting in a slower electron flux and greater probability of ROS production when ATP production is upregulated (Erlanson‐Albertsson, 2003; Jastroch et al., 2010; Ricquier & Bouillaud, 2000; Speakman et al., 2004). This change in risk of ROS production was not associated with an increase in oxidative damage.

Muscle mitochondria also responded to mammary mass in lactating females, with state 3 respiration increasing and mRNA expression of PGC‐1α dropping (Wu et al., 1999) as mammary mass increased. Gutgesell et al. (2009) described a similar drop in PGC‐1α in skeletal muscle. This drop occurred in conjunction with a drop in PPAR‐α which regulates in fatty acid metabolism (Gutgesell et al., 2009). As the demand of the mammary gland increases, an increase in state 3 respiration should result in skeletal muscle cells having more efficient population of mitochondria that use less fatty acids. Additional measurements of NRF‐1 and NRF‐2 would be beneficial in future studies to untangling the effects of PGC‐1α on substrate metabolism versus mitochondrial biogenesis. This addition would allow investigators to confirm that mitochondrial biogenesis is also downregulated during lactation (Gleyzer, Vercauteren, & Scarpulla, 2005; Wu et al., 1999).

Both the liver and skeletal muscle of lactating mice displayed a reduction in select antioxidant protein levels, while others remained unchanged. Specifically, concentrations of two of the three antioxidants measured (catalase and CuZnSOD) in the liver mitochondria were lower, while lipid peroxidation was higher in lactating females compared to nonreproductive females, suggesting that downregulation of antioxidants was prioritized over protecting tissues from damage. Direct measurements of ROS should be conducted to confirm this effect. In skeletal muscle, one of the three antioxidants measured (catalase) was lower in lactating females compared to nonreproductive females. Cells rely on multiple antioxidant networks to protect them from free radical damage (Powers, Smuder, Kavazis, & Hudson, 2010). Our results cover multiple antioxidants expressed in the isolated mitochondria, the biomolecules closest to newly formed ROS. As a reduction in antioxidants has the potential to allow a greater percentage of existing free radicals to interact with and damage intracellular proteins, lipids, and mitochondrial DNA, the cost of downregulating antioxidants production is likely to provide an alternative benefit, such as making amino acids available for milk synthesis. These observations suggest that the substrate requirements for antioxidant production, mostly amino acids, are spared during lactation. Interestingly, even though the antioxidant MnSOD did not differ between lactating and nonreproductive mice, MnSOD did increase with final litter size for the mice in the lactating group. This finding suggests antioxidant systems, and likely ROS production, are not entirely downregulated during lactating.

Relative expression of UCP2 mRNA in liver and muscle displayed high variation among mice in both in the lactating and nonreproductive groups. In lactating animals, variation was explained, in part, by variation in the demand on females. Interestingly, this response to demand varied by organ. Liver UCP2 was positively correlated with mammary mass while muscle UCP2 was negatively correlated with both mammary mass and litter size. High expression of UCP2 in the liver of lactating females with high mammary mass will act to reduce the proton motive force and limit the elevated level of ROS indicated by increased lipid peroxidation (Ricquier & Bouillaud, 2000). Thus, higher UCP2 in the liver of lactating females can be assumed to partially compensate for low antioxidant production. UCP's are a direct target of PGC‐1α (Gutgesell et al., 2009). Thus, the reduction in muscle UCP2 in skeletal muscle may have been a direct response to a decline in PGC‐1α muscle activity. UCP3 is more abundant in muscle than UCP2, and there is strong evidence that UCP3 is typically downregulated in skeletal muscle at peak lactation (Xiao, Grove, & Smith, 2004). A reduction in UCP3 has been proposed to reduce relative heat generation associated with proton moment across the inner mitochondrial membrane (Xiao, Grove, & Smith, 2004), and it is possible that a drop in UCP2 provides a similar function (Schrauwen & Hesselink, 2002). Heat dissipation has been proposed to be a constraint on the ability to allocate resources to reproduction (Speakman & Krol, 2005). Nevertheless, the contribution to UCP2 and UCP3 to heat dissipation remains controversial, and a drop in these proteins in muscle may be equally valuable in sparing amino acid substrate for competing demands.

When considered in total, our findings suggest that the liver and muscle of lactating mice display both commonalities and differences in their energetic responses to lactation. Adapting to relative demand, the skeletal muscle of lactating females appear to more efficiently produce ATP and avoid using fatty acids, while the liver meets its elevated demand largely by increasing size, rather than adjusting the bioenergetic capacity of its mitochondria. Both organs displayed reduced antioxidant protein levels, which could allow amino acids that would otherwise be used for antioxidant production to be used for the synthesis of milk proteins, including milk caseins and whey. Change in how amino acids are allocated to different processes, such as antioxidant synthesis, could be verified by feeding females isotopically labeled amino acids (Zera, Potts, & Kobus, 1998) and comparing the destination of these amino acids in lactating versus nonreproductive mice.

Our observations were not entirely consistent with observations in prior studies that evaluated mitochondrial function at peak lactation in house mice. These discrepancies suggest that changes in mitochondrial function with life events should not be assumed and may vary with environment and possibly the history of the individual or lineage. When kept in standard laboratory rodent boxes, Pichaud, Ballard, Tanguay, and Blier (2013) found that ETS activity (uncoupled respiration induced with 4‐(trifluoromethoxy)phenylhydrazone, i.e., FCCP) decreased in the liver of female house mice at peak lactation. This value is functionally similar to state 3 respiration described herein, which did not differ between groups. In a companion study, Garratt, Pichaud, King, and Brooks (2013) also found that oxidative damage (protein carbonyls) was lower and antioxidant levels were higher in the liver during lactation, which is counter to our findings. In laboratory mice, Zheng, Lin, Zheng, Cao, and Zhao (2015) found a reduction in ROS generation (H_2_O_2_), lipid peroxidation (malondialdehyde), and glutathione peroxidase activity in muscle and no change in ROS, a reduction in lipid peroxidation, and an increase in total antioxidant capacity in skeletal muscle in lactating laboratory mice versus nonreproductive mice. It is worth noting that we targeted lipid damage and antioxidant production in isolated mitochondria, whereas as Garratt et al. (2013) and Zheng et al. (2015) used homogenized tissue and targeted damage to proteins. As catalase and CuZnSOD are abundant in the cytosol and peroxisomes, in addition to being present in the mitochondria, it is possible that that intra‐ and extramitochondrial level of these antioxidants display different responses to lactation (Higashi & Peters, 1963; Sturtz, Diekert, Jensen, Lill, & Culotta, 2001). Interestingly, when housed in larger enclosures, reproductive female house mice (Garratt et al., 2011) also display lower liver oxidative damage compared to nonreproductive individuals, but this decrease disappeared when reproductive females were forced to contend with territorial intrusion. Compared to the mice in Garratt et al. (2011), the mice described herein had the opportunity to travel even greater distances, to experience more social interactions with other members of their deme, and to contend with natural changes in ambient conditions and exposure to the sounds of predators, such as the Eastern Screech Owls (*Megascops asio*) near our mouse facility. Environmental conditions and social interactions likely play an important role in determining the bioenergetic profiles of animals during reproduction and may help to explain the high variation in the relative accumulation of oxidative damage between species and among studies within species (Blount et al., 2016; Speakman & Garratt, 2014; Speakman et al., 2015). In this study, the addition of enclosure as a random effect increased standard error for several variables (Hood personal observation). It is possible that differences in social conditions were responsible for variance among enclosures. Also, nonreproductive animals were maintained independent of males and thus, the social environment of these mice would have differed from that of the lactating mice. Future studies should consider including vasectomized males with nonreproductive females.

We selected liver and muscle in this study not only because of their high‐energy demand but also because of their large size, which allowed us to complete all of the measurements described herein. To recover enough mitochondria to measure mitochondrial respiratory function, our techniques required at least 0.3 g of fresh tissue. Future studies should consider additional organs and alternative methods for evaluating mitochondrial respiratory function. It would be particularly interesting to determine whether changes in mitochondrial function are responsible for the purported trade‐off between reproduction and immune function observed in some species of mammals (Fló et al., 1994; Medina et al., 1993) and other taxa (Norris & Evans, 2000).

## 5 Conclusions and Future Directions

Our findings show that variation in the function of mitochondria play a role in allowing organs to partition available resources during reproduction, a response that should maximize milk production. While several prior studies have evaluated the oxidative state of the animals by evaluating the accumulation of the products of damage in blood, our results highlight that organs may respond differently to an energetically demanding event and that such differences will be overlooked without quantifying the status of individual organs. Future studies should evaluate how organs respond to differences in the relative demand of reproduction and evaluate the role that a female's environment plays in bioenergetic processes, such as those described herein.

Following the free radical theory of aging, it is possible that oxidative damage detected during reproduction could contribute to a reduced capacity of the liver, particularly if that damage is not repaired, and if oxidative damage is a persistent feature of reproduction. To address such questions, it is vital to characterize the state of the liver or other damaged organs after the reproductive event has ended to determine whether the observed damage is persistent or whether it largely disappears and the liver and reproduction organs return to nonreproductive size and function once lactation has ended (Zhang & Hood, 2016).

## Conflict of Interest

None declared.
